# Effectiveness of the GumChucks flossing system compared to string floss for interdental plaque removal in children: a randomized clinical trial

**DOI:** 10.1038/s41598-020-59705-w

**Published:** 2020-02-20

**Authors:** Joshua Lin, Márcia Dinis, Chi-Hong Tseng, Melissa Agnello, Xuesong He, Daniela R. Silva, Nini C. Tran

**Affiliations:** 1 University of California, Los Angeles School of Dentistry | Section of Pediatric Dentistry, California, USA; 2 University of California, Los Angeles | Department of Medicine, Statistics Core, California, USA; 3 University of California, Los Angeles School of Dentistry | Section of Oral Biology, California, USA; 4 The Forsyth Institute| Microbiology, Cambridge, USA

**Keywords:** Oral hygiene, Paediatric dentistry, Minimal intervention dentistry

## Abstract

Flossing, an important oral hygiene skill, is technique-sensitive and challenging for children with developing manual dexterity. GumChucks is a novel flossing device designed to assist children with proper flossing technique. The aim of this study was to assess the efficacy of the GumChucks flossing device compared to string floss (SF). We conducted a randomized trial with 40 children aged 4–15 years at the UCLA Children’s Dental Center from January- April 2017. Participants were randomly assigned to either GumChucks or SF. Interdental plaque score (IPS) and gingival index (GI) were recorded at baseline and 4-week post-usage. Flossing speed and interdental plaque reduction were also determined immediately after first use. In addition, questionnaires were completed by children, parents and dentists. Overall, children flossed significantly faster (*p* < 0.001) and achieved greater IPS reduction after first use (47.0% vs. 26.8%) with GumChucks compared to SF. After 4-week post-usage, children ages 10–15 in the GumChucks group demonstrated significantly greater improvement in GI and IPS from baseline (*p* < 0.01) and greater efficacy in interdental plaque removal compared to the SF group (*p* < 0.01). Children ages 4–9 flossed more effectively (*p* < 0.01) with GumChucks after first use, but no significant IPS and GI improvement after 4-week post-usage. Children preferred GumChucks (92.5%) over SF, with a similar positive attitude reported by parents and dentists. GumChucks is an effective alternative interdental plaque removal aid that allows children to floss with greater speed and efficacy, with recommended parental supervision for children under age 10.

## Introduction

Oral health is an integral part of the overall individual’s general health. Dental caries and gingivitis are two common oral health problems in childhood. The prevalence of dental caries can be detected throughout childhood stages with 23% in children ages 2–5, 21% in children ages 6–11, and 58% in adolescents ages 12–19^1^. Though the prevalence of gingivitis is uncommon in the early stage of primary dentition, it increases to approximately 50% in children ages 4–5 and continues to increase with age until nearly 100% at puberty^2^. Children’s oral health condition is an important determinant of their subsequent general and oral health outcomes as adults. Therefore, it is of utmost importance to establish proper oral hygiene habits early in life to prevent detrimental effects to permanent dentition and adult periodontium.

It is well established that the presence of bacterial plaque plays an important role in the development and progression of dental caries and gingivitis^3–8^. However, interdental sites pose additional risks to the susceptibility of dental caries and gingivitis. Plaque build-up at interproximal sites was shown to be more acidogenic than in other areas of the oral cavity^9^. Additionally, interdental contacts between primary teeth allow favorable bacterial growth because the contact areas are not as tight as those between permanent teeth^10^.

Flossing is a means to mechanically remove and disrupt the complex architecture of interproximal plaque^11^. Previous studies have suggested that flossing as an adjunct to toothbrushing is more effective in interproximal plaque removal than brushing alone in adults^12–14^. Similar investigations in children are limited, and most demonstrated that a combination of toothbrushing and different flossing devices did not produce significant clinical improvement^15–20^. The unappreciable results may be attributed to the usability and design of these flossing methods, which are challenging for children’s manual dexterity.

Flossing is a technique-sensitive process and is challenging for children with developing fine motor skills. As such, several guidelines have been proposed to address the unique needs of the child population and to provide general recommendations for the management of children’s oral health. The American Academy of Pediatric Dentistry (AAPD) recommends that oral hygiene is initially the responsibility of the parent, and that home care should be performed jointly by the parent and child until the child demonstrates the ability to perform personal hygiene techniques alone^21^. Pertaining to the flossing regimen, the AAPD recommends that while initially parents should floss or supervise flossing for their children, they should master the flossing skill by the age of 10^22^. Similar oral hygiene guidelines for children are also recommended by the Academy of Pediatrics (AAP)^23^.

**Figure 1:**
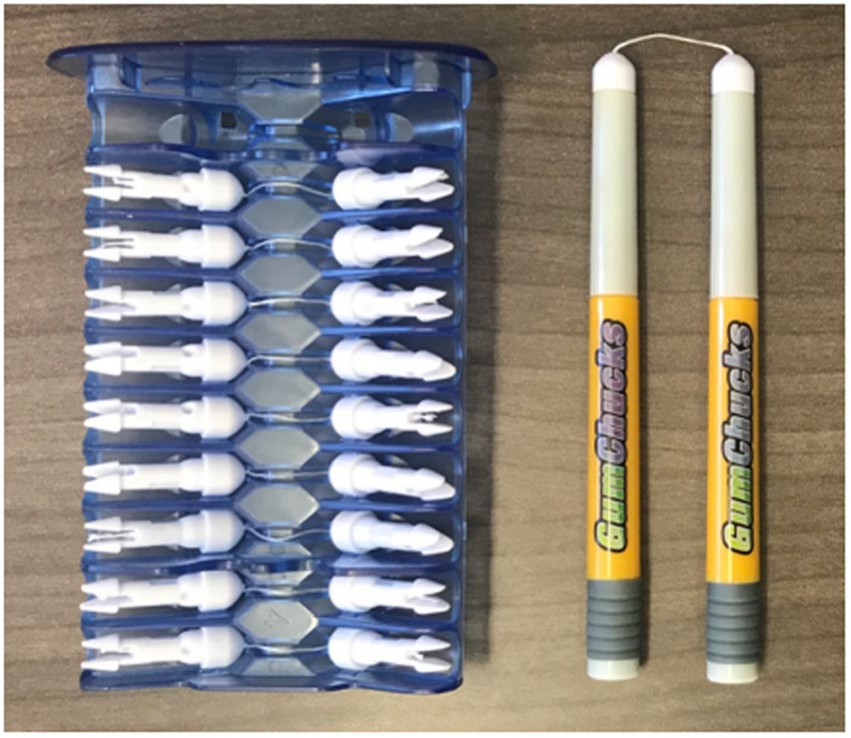
GumChucks flossing device used in the study.

“C-shape” flossing is generally considered as the proper flossing technique. Following this method, the floss is gently passed between teeth and is curved into a C shape against the side of one tooth when the floss reaches the gum line^24^. This flossing technique can be difficult for children to perform when using a conventional string floss. Although the importance of proper flossing is well established, there is a lack of flossing devices that facilitate children to floss more effectively. GumChucks is an innovative flossing system resembling mini nunchucks. The two-handle design eliminates the need to wrap the floss around fingers, and a proper flossing technique is facilitated by the nature of its curved shaped as shown in Fig. 1. The purpose of this study is to evaluate the interdental plaque removal efficacy, flossing speed, and reduction in gingival inflammation of a new flossing system (GumChucks) compared to string floss, and to assess preference between the two flossing products in children, parents and dentists.

## Material and Methods

This study was approved by the University of California Los Angeles Institutional Review Board (#16-000617). The study was conducted between January 2017 and April 2017. It was registered at ClinicalTrials.gov with a registration number of NCT04057430 and date of August 13, 2019. The study was conducted and reported in accordance with the declaration of Helsinki for Biomedical Research Involving Human Subjects.

### Study population

Participants were recruited from the pediatric patient population of the University of California, Los Angeles (UCLA) Children’s Dental Center. Participants were deemed eligible based on the following inclusion criteria: (1) healthy children aged 4–15 years, who were not on any type of medication; (2) presence of at least 4 posterior interdental contacts; (3) ability to follow verbal and/or written instruction; (4) availability for a 4-week study period; and (5) one parent or legal guardian of each included child participant. Participants were excluded from the study if they (1) had antibiotics within the last 6 months, (2) were pregnant. A convenience sample of 30 practicing pediatric dentists and general dentists, who provide dental care for children and who had no knowledge of GumChucks flossing product prior to participating in the study were included. Parents or legal guardians of all participants signed written informed consent prior to the commencement of the study.

### Study design

**Figure 2:**
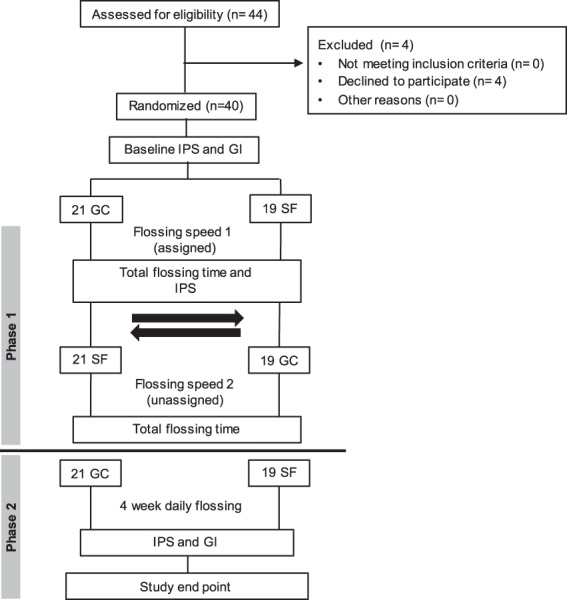
Study design and participant flow chart. GC = GumChucks; SF = String floss; IPS = Interdental plaque scores; GI = Gingival index.

This was a 2-phase randomized clinical study comparing the efficacy of GumChucks versus string floss (Oral-B Glide Pro-Health Original Floss). The study design and participant flowchart is shown in Fig. 2. At the baseline visit, all participants (child participants, parents of child participants, and dentists) viewed an instructional video about proper flossing technique (C-shape flossing) and GumChucks usage. Child participants and their parents were also demonstrated and supervised how to correctly use both flossing products and proper brushing technique. Only child participants participated in the 2-phase clinical study. Random allocation of participants into the two investigative groups was performed using the random number generator formula (“ = rand”) in Excel (Microsoft, Inc). Phase I was an intra-individual cross-over study in which all participants used both flossing products in order to assess the flossing speed of each, and to evaluate the efficacy of the assigned flossing product in plaque removal immediately after use. For Phase II, participants were given adequate supply and instructed to use the assigned flossing product daily for four weeks in conjunction with routine toothbrushing practice (no other interdental cleaning product). For compliance, participants were encouraged to keep records of their flossing routine, and were given contact information in the event that participants needed guidance regarding flossing technique or needed additional flossing product. Participants were also asked to return any unused floss product.

Preferences of child participants, their parents and dentists toward the use of GumChucks in comparison to string floss was measured by a 5-point Likert scale questionnaire. Child participants and their parents completed questionnaires after using both flossing products at the end of Phase I study, while dentists completed questionnaires after viewing the video and/or live demonstration of GumChucks usage on a model of the oral cavity.

### Study outcomes

Clinical parameters evaluated were the gingival index (GI), interdental plaque scores, and flossing speed. GI^25^ and interdental plaque scores^26^ were evaluated only at the interdental sites expected to be involved during a proper flossing technique. First, gingival inflammation was assessed using the GI index, in which scores were taken of the buccal and lingual margins of the interdental papillae adjacent to interdental contacts. GI scores were averaged for each child by summing the score from each individual surface and dividing by total number of surfaces. GI was measured at baseline and 4 weeks. Interdental plaque scores were recorded after the teeth were stained with plaque disclosing solutions (GUM Red-Cote) for 30 seconds followed by a water rinse. Interdental plaque was scored as either presence or absence at 4 sites of each tooth (mesio-buccal, disto-buccal, mesio-lingual, and disto-lingual). The interdental plaque score for each participant is the sum of all sites stained positive with disclosing solution divided by the total number of sites assessed. Interdental plaque scores were measured at baseline and immediately post-usage, and after 4 weeks of daily usage of the assigned flossing product.

To assess the flossing speed of each floss product, the total time for a participant to floss all interdental contacts was measured in seconds, starting with the assigned flossing product. All participants were given instructions on c-shape flossing with both flossing products prior to the speed test. Participants were instructed to floss in front of a mirror.

All assessments were performed by an examiner team of three trained pediatric dental residents assisted by data recorders. The examiners were calibrated for consistency using three subjects not participating in the study by assessing GI and interdental plaque scores of these subjects. Correlation coefficients for intra-examiner reproducibility ranges from 0.95 to 1.00 for GI and 1.00 for interdental plaque scores. For inter-examiner agreement, at least two examiners are in agreement for all GI score and all three examiners are in agreement for 83% of the interdental plaque scores. The Fleiss’ kappa coefficient for inter-examiner agreement was 0.57 for GI and 0.80 for interdental plaque scores. Examiners and data recorders were not aware of the participant’s group assignment at 4-week post usage. However, the blinding of examiners during the assessment of the flossing speed and interdental plaque scores immediately after flossing was not feasible.

### Sample size and power analysis

The sample size was estimated to be 360 interdental sites in each group (18 interdental sites per participants x 20 participants). With this sample size, there is 80% statistical power to detect a difference of 0.35 in effect size, which is about 8.2% interdental plaque reduction between GumChucks and string floss group at significance level of 0.05.

### Statistical analysis

Inter-individual differences in flossing speed between the two devices were assessed with paired t-test. Interdental plaque scores and gingival indices were defined for each participant as the average score over all tooth surfaces. Differences in mean baseline scores vs. scores at 4 weeks within each treatment group were compared using paired t-tests. To compare between treatment groups, we calculated the average percent change in IPS and GI in each group, and assessed significance using unpaired t-tests. Subgroup analyses were performed in children ages 4–9 years old and ages 10–15 years old. GraphPad Prism v.7 was used for t-tests. Fleiss’ kappa was calculated with R software (version 3.5.0). Statistical significance was defined as *p ≤ 0.05; **p ≤ 0.01; ***p ≤ 0.001.

## Results

### Participant demographics

Forty children aged 4–15 years old, who were pediatric patients of the University of California, Los Angeles (UCLA) Children’s Dental Center, 40 parents of child participants, and 30 dentists were enrolled in the study. 44 children were initially screened and deemed eligible, and 4 subsequently declined to participate (Fig. 2). 40 child participants completed the 2-phase randomized clinical trial. Participants’ age, gender, total numbers of interdental sites, and group assignments are shown in Table 1. Of the total child participants, 18 (45%) were male and 22 (55%) were female. The average age was 9.8 (±2.7) years with an age range of 4–15 years. After randomization, 21 participants were assigned to the GumChucks group (average age of 10.3 ± 2.1 years) and 19 were assigned to the string floss group (average age 9.3 ± 3.2). A total of 879 interdental sites was included in the study, with an average of 22.0 (±3.0) interdental sites per individual.

**Table 1:** Age, Gender, and Interdental Site Data of Child Participants.

Group	No.	Males (%)	Females (%)	Mean Age (SD)	Total no. of interdental sites	Mean no. of interdental sites (SD)
All Children	40	18	22	9.8 ± 2.7	879	22.0 ± 3.0
GumChucks	21	8	13	10.3 ± 2.1	465	22.1 ± 3.5
String floss	19	12	7	9.3 ± 3.2	414	21.8 ± 2.4
4–9 years old	16	7	9	7.1 ± 1.8	324	20.3 ± 2.1
GumChucks	7	1	6	8.0 ± 1.5	143	20.4 ± 2.4
String floss	9	6	3	11.4 ± 1.3	181	20.1 ± 1.9
10–15 years old	24	13	11	11.6 ± 1.5	555	23.1 ± 3.0
GumChucks	14	7	7	11.4 ± 1.3	322	23.0 ± 3.8
String floss	10	6	4	11.8 ± 1.7	233	23.3 ± 1.8

### Clinical parameters

**Figure 3:**
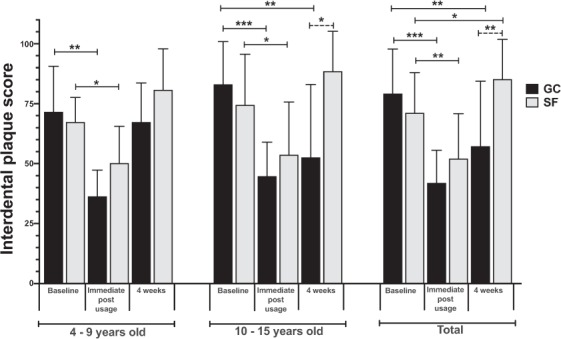
Interdental plaque scores. Comparison of interdental plaque scores at baseline, immediately post-usage, and after 4-week post-usage between GumChucks (GC, black bars) and string floss (SF, grey bars) in children aged 4–9 years old, 10–15 years old, and total child participants. Statistical significance was assessed using paired t-tests within treatment groups. Inter-group comparison was performed on mean percent reduction in interdental plaque scores using unpaired t-test (indicated with dashed line). Significance is indicated as follows: *p ≤ 0.05; **p ≤ 0.01; ***p ≤ 0.001.

Comparison of mean interdental plaque scores at baseline, immediately post-usage, and after 4 weeks for GumChucks and string floss groups is shown in Fig. 3. Statistical analysis revealed no significant baseline differences between the GumChucks and string floss groups for all children. Immediately after use, both flossing products significantly decreased interdental plaque scores relative to baseline, with a greater mean reduction in GumChucks than string floss group for all children (47.0%; *p* < 0.001 versus 26.8%; *p* < 0.01), age 4–9 years (49.1%; *p* < 0.01 versus 25.5%; *p* < 0.05), and age 10–15 years (46.1%; *p* < 0.001 versus 27.9%; *p* < 0.05). At 4-week post-usage, overall, GumChucks significantly decreased mean interdental plaque scores in children (*p* < 0.01) and provided 27.7% plaque reduction relative to baseline. We further performed subgroup analyses of children 4–9 years old (n = 16, average age 7.1 ± 1.8 years) and children 10–15 years old (n = 24, average age 11.6 ± 1.5 years). Children aged 10–15 years in the GumChucks group achieved significant mean interdental plaque reduction of 36.6% relative to baseline (*p* < 0.01), while children aged 4–9 years showed minimal and not significant interdental plaque reduction of 5.8% (*p* = 0.70). In contrast, children in the string floss group demonstrated a significant increase in the mean interdental plaque scores of 19.9% relative to the baseline (*p* < 0.05). Comparing between GumChucks and string floss, there was no significant difference in interdental plaque removal efficacy immediately after usage. Importantly, at 4-week post usage, GumChucks was significantly more effective in the removal of interdental plaque than string floss in all children (*p* < 0.01).

**Figure 4:**
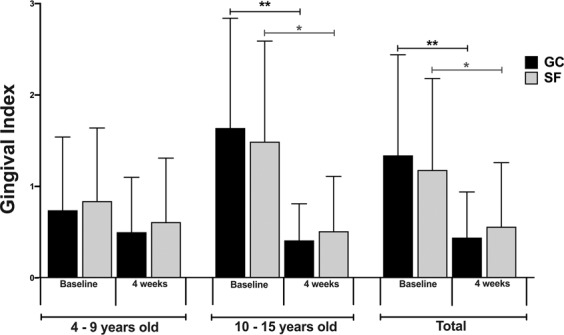
Gingival index scores. Comparison of gingival index scores at baseline and after 4-week post-usage between GumChucks (GC, black bars) and string floss (SF, grey bars) in children aged 4–9 years old, 10–15 years old, and total child participants. Statistical significance was assessed using paired t-tests within groups and unpaired between groups and is indicated as follows: *p ≤ 0.05; **p ≤ 0.01.

Gingival inflammation was assessed using the gingival index at baseline and 4-week post usage as shown in Fig. 4. At baseline, the mean gingival index scores did not differ significantly between groups (*p* > 0.05). Overall, after 4 weeks of daily usage, both GumChucks and string floss significantly lowered the mean gingival index score in all children (*p* < 0.01). However, in the age subgroups, significant improvement in gingival index scores was only observed in children aged 10–15 years (*p* < 0.01).

**Figure 5:**
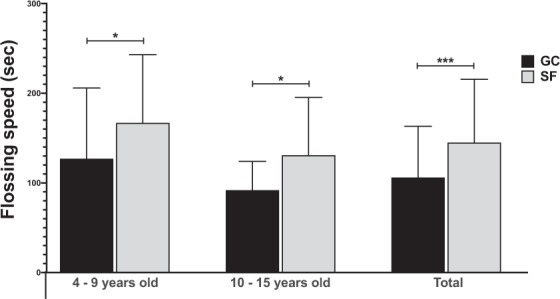
Flossing speed. Comparison of flossing speed, total time to floss all interdental contacts, between GumChucks (GC, black bars) and string floss (SF, grey bars) in children aged 4–9 years old, 10–15 years old, and total child participants. Statistical significance was assessed using paired t-tests and indicated as follows: *p ≤ 0.05; **p ≤ 0.01; ***p ≤ 0.001.

Flossing speed was also assessed to measure the total time for each participant to floss all interdental contacts with both flossing products (Fig. 5). Children aged 4–9 years, 10–15 years, and the entire child cohort flossed significantly faster with GumChucks versus string floss with an average speed of 127 s vs. 167 s (*p* < 0.05), 92 s vs. 131 s (*p* < 0.05), and 106 s vs. 145 s (*p* < 0.001), respectively.

### Preference for GumChucks compared to string floss

**Figure 6:**
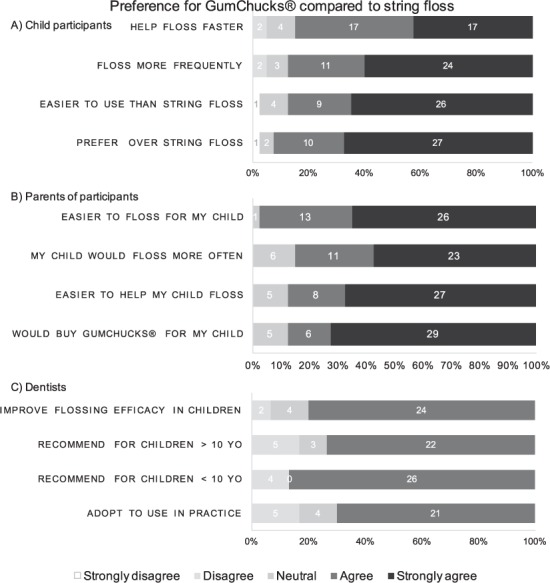
Preference for GumChucks vs. string floss. Selected questions and responses from questionnaires assessing preference towards GumChucks compared to string floss in (**A**) child participants, (**B**) parents of participants, (**C**) dentists.

Questionnaires were completed by child participants, their parents, and dentists to assess their preferences for GumChucks compared to string floss. Selected questions and respondent data are shown in Fig. 6, which includes the numbers and the percentage of responses. Child participants agreed and strongly agreed that GumChucks would help them floss faster (85%), more frequently (87.5%), and are easier to use (87.5%). Notably, 92.5% of child participants reported that they would prefer to use GumChucks over string floss. Similarly, parents of child participants agreed and strongly agreed that GumChucks would be easier for their children to use (97.5%) and that they would floss more frequently (85%). 87.5% of the parents also reported that it would be easier for them to help their child floss with GumChucks, and they would buy GumChucks for their children to use.

Assessing dentists’ preference for GumChucks and string floss, 80% of respondents agreed that GumChucks would improve flossing efficacy in children regardless of age. Dentists would recommend GumChucks to children younger than 10 years old (86.7%) as well as children over the age of 10 (73.3%). Largely, 70% of dentists stated that they would adopt the use of GumChucks in their practice.

## Discussion

Flossing is widely accepted as a mechanical means to disrupt and remove interdental plaque, and an early preventive strategy for dental caries and gingivitis, two common oral health problems in children. It is also an important skill to develop during childhood as it may serve to establish valuable, life-long oral hygiene habits. C-shape flossing, a proper flossing technique, is challenging for children with developing manual dexterity to perform with conventional string floss. Currently, the AAPD recommends that parents should floss or supervise flossing for their children up until the age of 10 to ensure proper flossing. Limited research investigating the effectiveness of flossing devices has been conducted in children, and most found no additional benefit when comparing to brushing alone, suggesting the need for an alternative flossing method to assist children in the removal of interdental plaque and maintaining oral health.

This study indicates that children were able to remove interdental plaque more effectively, decrease gingival inflammation and floss faster with GumChucks in comparison to string floss. Of note, after first use, children aged 4–9 years were able to achieve comparable reduction in interdental plaque with children aged 10–15 years (49.1% versus 46.1%), suggesting that, with proper instructions, this age group can floss effectively using GumChucks. After 4 weeks of usage, GumChucks also demonstrated greater interdental plaque reduction than string floss, but only in children aged 10–15 years. While the efficiency in interdental plaque removal of the floss is an important measurement, the reduction in gingival inflammation is clinically relevant. Both GumChucks and string floss significantly decrease gingival inflammation in children aged 10–15 years after 4 weeks of usage. This result, however, is unexpected as children in the string floss group were shown to have a significant increase in mean interdental plaque scores from the baseline. This could be due to the fact that GI does not always positively correlated with plaque scores^27^. In terms of total flossing time, children flossed about 25% faster with GumChucks compared to string floss (106 s vs. 145 s). This is less than the two-minute brushing time generally recommended by oral health professionals. In addition, the questionnaire data revealed a strong preference towards GumChucks by children, parents and dentists.

The results of this study are in agreement with a previous study in an Indian population of children ages 6–12 in which reduction of plaque and increased flossing frequency was observed with the use of GumChucks compared to standard floss^28^. Studies in adult populations have demonstrated a reduction in clinical parameters and preference toward flossing devices compared to conventional finger flossing^29–32^. In contrast, a study in children concluded that a combination of toothbrushing and different flossing devices (finger-floss, looped-floss and floss-holders) did not produce significant clinical improvement in comparison to toothbrushing alone in third grade schoolchildren^20^. The impact of flossing devices may be underestimated in the latter study since both free and interproximal sites were assessed together for clinical indices^33^.

This study contributes a clinical investigation in children to the existing body of research comparing the efficacy between flossing modalities. The strength of our study is the investigative question assessing whether children under the AAPD recommended age would be able to floss independently and effectively with a new flossing device (GumChucks) compared to a conventional string floss. In addition to providing evidence to support an effective flossing alternative to assist children with developing manual dexterity, our results suggest an effective supplementary flossing tool for the current oral hygiene guidelines for children. We also incorporated an intra-individual study to assess the flossing speed and efficacy in interdental plaque removal after first use. This design lessens the variable effect of participants’ manual dexterity and motivation on the outcomes of total flossing time and interdental plaque scores after first use^34^. Additionally, our examiners were calibrated for consistency, and the clinical parameters were assessed only at the interproximal sites where the effect of the flossing devices is most correlated.

This study is not without limitations. First, the total sample size for this study is 40 children. This sample size gave adequate power for the present comparison study; however, in the age subgroups, the statistical power was reduced with less numbers of children in 4–9 age group. This could potentially explain why the reduction in the interdental plaque in this age group was not significant at 4-week post usage. The duration of this study was 4 weeks without the addition of the intermittent basic hygiene period. This length of study time may not be sufficient to allow the establishment of stable clinical parameters and the comparison of these clinical parameters between basic hygiene and experimental period^34^. As mentioned in material and methods, the blinding of examiners during the assessment of the flossing speed and interdental plaque scores immediately after use was not feasible as they were present to ensure that each participant flossed properly. This may be a potential source of bias in the clinical outcomes.

Caution should be exercised when interpreting data from this study. The significant improvement in gingival condition and interdental plaque scores in the GumChucks group compared to string floss could come from the motivation effect of children^34^. Children in GumChucks group may be motivated by kid-friendly packaging that also included a picture of C-shape flossing and an encouraging quote to develop a healthy flossing habit. It is also important to note that children in the 4–9 age group had a gingival index score equivalent to mild inflammation at baseline. Therefore, appreciable improvement may be clinically detected, but not statistically significant.

The present study provided evidence that children over 10 years old are able to floss more effectively, with less time, and have better improvement in gingival inflammation with GumChucks compared to the conventional string floss after 4-week of usage. Findings also suggested that children under 10 years old can floss faster and potentially more effectively with GumChucks over time. However, a larger and longer confirmatory study is needed. Parental supervision is recommended for children under the age of 10 when using GumChucks. In this study, children and their parents reported preference for GumChucks over string floss. In an ongoing study with an adult population, greater efficacy in the removal of interdental plaque is also observed after first use compared to string floss. GumChucks could be an effective alternative to string floss to establishing flossing habits and maintaining oral health not only for children but for the entire family.

## References

[1] Dye, B. A., Thornton-Evans, G., Li, X. & Iafolla, T. J. Dental caries and sealant prevalence in children and adolescents in the United States, 2011–2012. *NCHS Data Brief*, 1–8 (2015). 25932891

[2] Griffen, A. Gingivitis and periodontitis in children and adolescents. *UpToDate*, https://www.uptodate.com/contents/gingivitis-and-periodontitis-in-children-and-adolescents (2019).

[3] Selwitz RH, Ismail AI, Pitts NB (2007). Dental caries. Lancet.

[4] Aas JA (2008). Bacteria of dental caries in primary and permanent teeth in children and young adults. J. Clin. Microbiol..

[5] Caufield, P. W. & Griffen, A. L. Dental caries. An infectious and transmissible disease. *Pediatr Clin North Am* 47, 1001–1019, v (2000). 10.1016/s0031-3955(05)70255-8 11059347

[6] Socransky SS (1970). Relationship of bacteria to the etiology of periodontal disease. J. Dent. Res..

[7] Slots J, Listgarten MA (1988). Bacteroides gingivalis, Bacteroides intermedius and Actinobacillus actinomycetemcomitans in human periodontal diseases. J. Clin. Periodontol..

[8] Silness J, Loe H (1964). Periodontal Disease in Pregnancy. Ii. Correlation between Oral Hygiene and Periodontal Condtion. Acta Odontol. Scand..

[9] Igarashi K, Lee IK, Schachtele CF (1989). Comparison of *in vivo* human dental plaque pH changes within artificial fissures and at interproximal sites. Caries Res..

[10] Pari A, Ilango P, Subbareddy V, Katamreddy V, Parthasarthy H (2014). Gingival diseases in childhood - a review. J. Clin. Diagn. Res..

[11] Hill, I. C. C. & Locust, N. J. 07760), White, Robert D. (65 Glen Gray Rd., Oakland, NJ, 07436). Method of treating the oral cavity with dental floss containing chemotherapeutic agents. United States patent (1992).

[12] Terezhalmy GT, Bartizek RD, Biesbrock AR (2008). Plaque-removal efficacy of four types of dental floss. J. Periodontol..

[13] Kiger RD, Nylund K, Feller RP (1991). A comparison of proximal plaque removal using floss and interdental brushes. J. Clin. Periodontol..

[14] Hill HC, Levi PA, Glickman I (1973). The effects of waxed and unwaxed dental floss on interdental plaque accumulation and interdental gingival health. J. Periodontol..

[15] Granath LE (1979). Intraindividual effect of daily supervised flossing on caries in schoolchildren. Community Dent. Oral. Epidemiol..

[16] Halla-Junior R, Oppermann RV (2004). Evaluation of dental flossing on a group of second grade students undertaking supervised tooth brushing. Oral. Health Prev. Dent..

[17] Horowitz AM (1979). A comparison of available strategies to affect children’s dental health: primary preventive procedures for use in school-based dental programs. J. Public. Health Dent..

[18] Horowitz AM, Suomi JD, Peterson JK, Lyman BA (1977). Effects of supervised daily dental plaque removal by children: II. 24 months’ results. J. Public. Health Dent..

[19] Horowitz AM, Suomi JD, Peterson JK, Voglesong RH, Mathews BL (1976). Effects of supervised daily dental plaque removal by children: first-year results. J. Public. Health Dent..

[20] Rich SK, Friedman JA, Schultz LA (1989). Effects of flossing on plaque and gingivitis in third grade schoolchildren. J. Public. Health Dent..

[21] American Academy of Pediatric Dentistry (2005). Guideline on adolescent oral health care. Pediatr. Dent..

[22] American Academy of Pediatric Dentistry. Fast facts, https://www.aapd.org/assets/1/7/FastFacts.pdf (2013).

[23] American Academy of Pediatrics. A Pediatric Guide to Children’s Oral Health, https://www.aap.org/en-us/advocacy-and-policy/aap-health-initiatives/Oral-Health/Documents/OralHealthFCpagesF2_2_1.pdf (2009).

[24] American Dental Association. 5 Steps to a Flawless Floss, https://www.mouthhealthy.org/en/az-topics/f/flossing-steps (2018).

[25] Loe H, Silness J (1963). Periodontal Disease in Pregnancy. I. Prevalence and Severity. Acta Odontol. Scand..

[26] Ong G (1990). The effectiveness of 3 types of dental floss for interdental plaque removal. J. Clin. Periodontol..

[27] De David SC (2018). Correlation between plaque control and gingival health using short and extended oral hygiene intervals. Clin. Oral. Investig..

[28] Kiran SD (2018). Comparison of Plaque Removal Efficacy of a Novel Flossing Agent with the Conventional Floss: A Clinical Study. Int. J. Clin. Pediatr. Dent..

[29] Barton RF, Diamond B (1980). Evaluation and patient acceptance of a mechanical dental flossing device compared to hand-held floss. Clin. Prev. Dent..

[30] Carter-Hanson C, Gadbury-Amyot C, Killoy W (1996). Comparison of the plaque removal efficacy of a new flossing aid (Quik Floss) to finger flossing. J. Clin. Periodontol..

[31] Mulligan R, Wilson S (1984). Design characteristics of floss-holding devices for persons with upper extremity disabilities. Spec. Care Dent..

[32] Spolsky VW, Perry DA, Meng Z, Kissel P (1993). Evaluating the efficacy of a new flossing aid. J. Clin. Periodontol..

[33] Lobene RR, Soparkar PM, Newman MB (1982). Use of dental floss. Effect on plaque and gingivitis. Clin. Prev. Dent..

[34] Kleisner J, Imfeld T (1993). Evaluation of the efficacy of interdental cleaning devices. How to design a clinical study. J. Clin. Periodontol..

